# Methylation Abnormalities in Mammary Carcinoma: The Methylation Suicide Hypothesis

**DOI:** 10.4236/jct.2014.514131

**Published:** 2014-12-01

**Authors:** Anne H. O’Donnell, John R. Edwards, Robert A. Rollins, Nathan D. Vander Kraats, Tao Su, Hanina H. Hibshoosh, Timothy H. Bestor

**Affiliations:** 1 Department of Genetics and Development, College of Physicians and Surgeons of Columbia University, New York, NY, USA; 2 Division of Genetics, Boston Children’s Hospital, Boston, MA, USA; 3 Center for Pharmacogenomics, Washington University School of Medicine, St. Louis, MO, USA; 4 Pfizer BioTherapeutics Research and Development, Center for Integrative Biology and Biotherapeutics, Pearl River, NY, USA; 5 Department of Pathology, College of Physicians and Surgeons of Columbia University, New York, NY, USA

**Keywords:** DNA Methylation, Mammary Carcinoma

## Abstract

Promoter silencing by ectopic *de novo* methylation of tumor suppressor genes has been proposed as comparable or equivalent to inactivating mutations as a factor in carcinogenesis. However, this hypotheses had not previously been tested by high resolution, high-coverage whole-genome methylation profiling in primary carcinomas. We have determined the genomic methylation status of a series of primary mammary carcinomas and matched control tissues by examination of more than 2.7 billion CpG dinucleotides. Most of the tumors showed variable losses of DNA methylation from all sequence compartments, but increases in promoter methylation were infrequent, very small in extent, and were observed largely at CpG-poor promoters. De novo methylation at the promoters of proto-oncogenes and tumor suppressor genes occurred at approximately the same frequency. The findings indicate that tumor suppressor silencing by *de novo* methylation is much less common than currently believed. We put forward a hypothesis under which the demethylation commonly observed in carcinomas is a manifestation of a defensive system that kills incipient cancer cells.

## 1. Introduction

It has long been known that genome demethylation is often an early step in multistage carcinogenesis [1], although the biological cause of this demethylation remains obscure and the role of demethylation in carcinogenesis is unknown. It has also been held that general demethylation is accompanied by focal *de novo* methylation targeted to CpG islands, and that *de novo* methylation of tumor suppressor CpG islands silences transcription of the gene in a manner that is functionally equivalent to an inactivating mutation [2]; this model will be referred to as the epimutation hypothesis.

There have been studies that called into question the importance of epimutation methylation in carcinogenesis. Smiraglia *et al*. [3] found that rates of *de novo* promoter region methylation could be several hundred-fold more common in tumor cells lines than in primary cancers. Close inspection of the literature in primary carcinomas yielded essentially no convincing examples of tumor suppressor methylation; in the large majority of cases, the reported *de novo* methylation occurs outside of promoter regions, and the proximal promoter is frequently not tested. This point is important because most sequences outside of proximal promoters are usually methylated to varying extents [4] [5] and this methylation has not been shown to affect expression. In addition, most studies that employ bisulfite sequencing and other PCR-based methods to map DNA methylation do not control the documented bias of these method in favor of methylated sequences [6].

Here we use Methyl-MAPS (methylation mapping by paired-end sequencing [4] [5]) to compare the genomic methylation patterns of a series of primary mammary carcinomas with normal breast tissues from the same subjects. Methyl-MAPS does not involve preselected primers or probes and yields high coverage, single-CpG resolution methylation profiles across the entire genome. The results confirm the reported variable loss of DNA methylation from all sequence compartments in carcinomas, but whole genome methylation profiling in primary mammary carcinoma indicates that the frequency of aberrant promoter methylation in this cancer is much lower than previously reported and may not play a major role in carcinogenesis. We suggest that the genome-wide demethylation that commonly occurs in mammary carcinoma may be a manifestation of a methylation-based anti-cancer defensive system, and that the silencing of tumor suppressor genes in carcinomas involves pathways other than *de novo* promoter methylation.

## 2. Results

**Figure 1:**
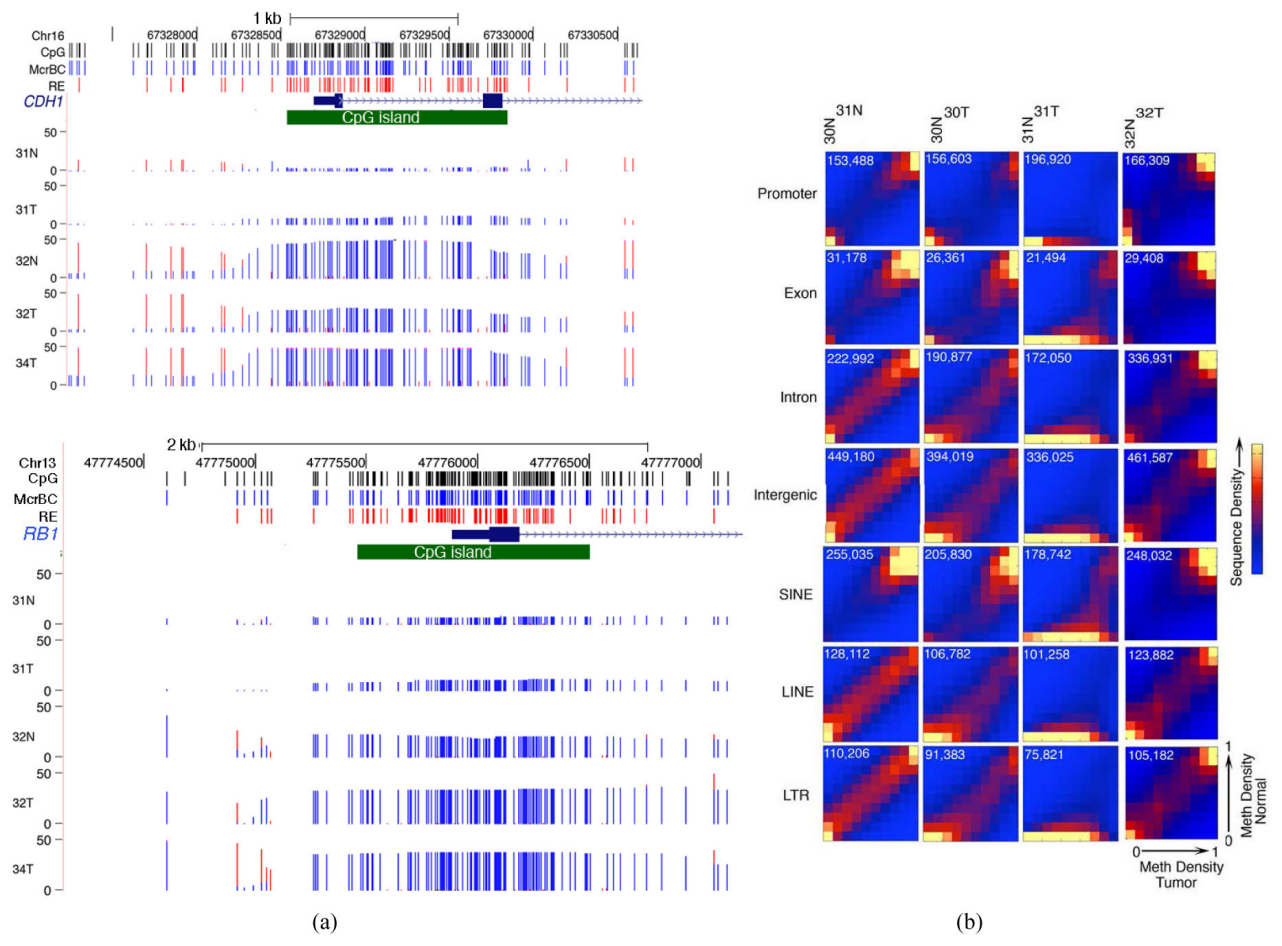
(a) Methyl-MAPS analysis of *CDH*1 and *RB*1 genes. Along the top of the figure, tick marks indicate locations of individual CpG sites (black), RE (red) and McrBC (blue) recognition sequences. Below the gene diagram, coverage of McrBC (blue bars) and RE (red bars) at cleavable sites is indicated as an overlayed histogram. The vertical scale indicates fold coverage over the range of 0 to 50×. The promoter-associated CpG island (green) for each gene can be seen to be unmethylated in all samples. (b) Global genome demethylation in mammary carcinoma. For each sample pair, the methylation levels of each CpG dinucleotide in control and cancer genomes was compared. The densities of the resulting scatter plots are shown as heat maps. Demethylation in a tumor relative to the normal is below and to the right the diagonal, while hypermethylation in a tumor relative to the normal is above and to the left of the diagonal. The number of CpGs analyzed in each sequence category is shown. Methylation patterns are largely conserved between the two normal tissues (30N and 31N) while 30T and 32T are partially demethylated and tumor 31T shows sweeping demethylation.

DNA was prepared from mammary ductal carcinomas that were judged by the study pathologist (H. H.) to contain >80% cancer cells Table S1. Methyl-MAPS was performed as described [4] [5]; more than 319 million paired-end reads were obtained, and these determined the methylation status of >2.7 billion CpG sites Table S2. The methylation status of the *CDH*1 and *RB*1 genes, two of the most commonly mutated genes in mammary carcinoma [7] is shown in Figure 1(a) and illustrates the type of primary data obtained with this approach. As is typical of genes whose promoters overlap a CpG island, the 5′ end of the gene is unmethylated, and methylation at other sequences in and around these genes is partial and variable [4] [5].

Genome-wide Methyl-MAPS data was compiled over multiple sequence categories and the methylation status of the tumor genomes was compared to that of DNA from normal adjacent breast tissues for tumors 30T, 31T, and 32T; only tumor DNA was analyzed from tumor 34T. Mean genome-wide fold coverage ranged from 4.9 to 60.1 Table S2. The results are shown in graphical format in Figure 1(b). The first column compares the methylation status of the two normal breast genomes where very strong concordance between the two samples is evident. The second through fourth columns compare the methylation status of tumor 30T to normal breast tissue 30N, tumor 31T to 31N and tumor 32T to 32N. Genome-wide demethylation was found to occur in all three tumors.

**Figure 2:**
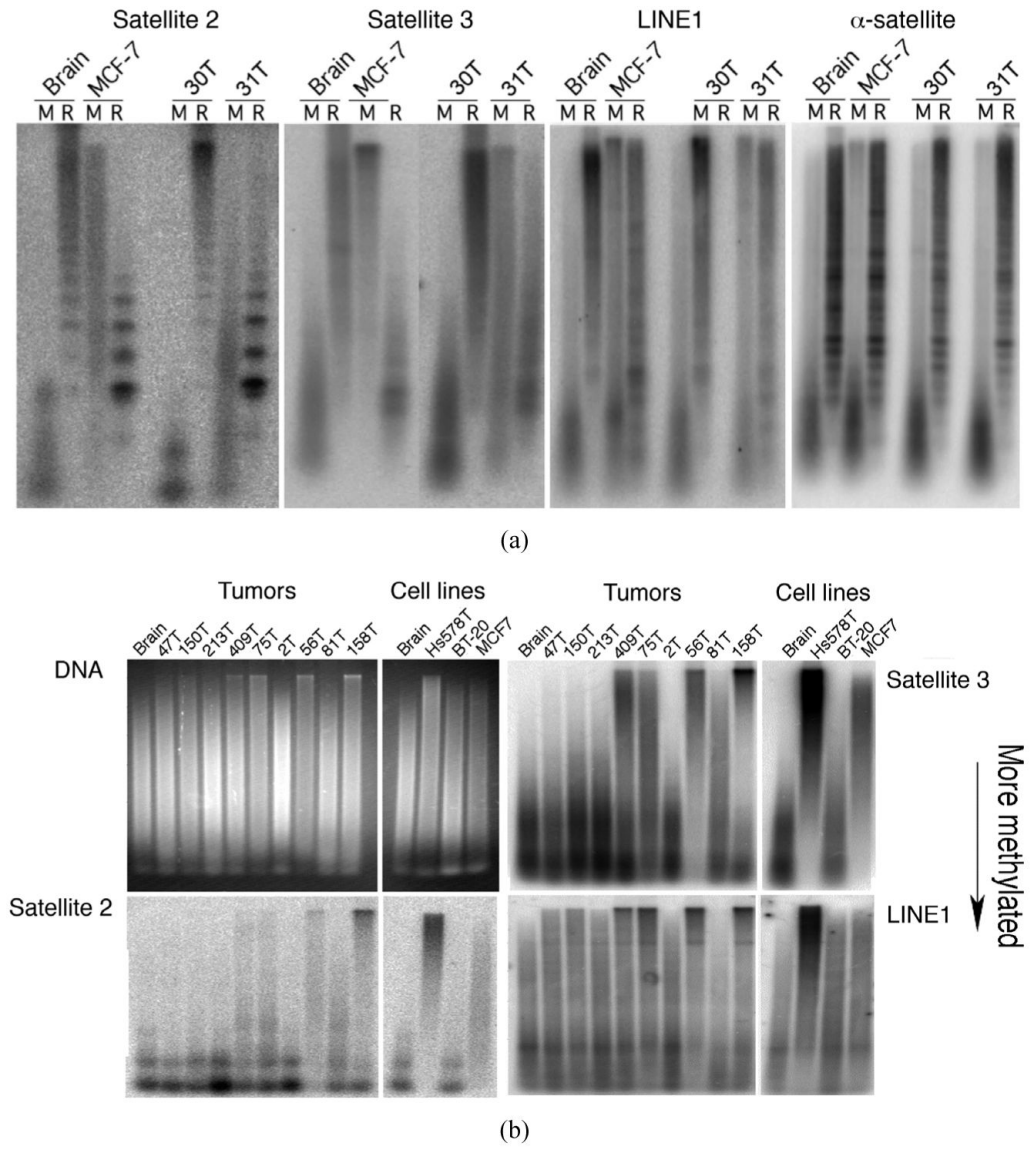
Genome demethylation in mammary carcinoma tumors and cell lines. (a) Resistance to digestion by the methylation-dependent endonuclease complex McrBC (M) indicates demethylation, while resistance to digestion by the RE (R) cocktail of methylation-sensitive endonucleases indicates methylation. MCF7, 30T and 31T show widespread hypomethylation in tandem (Satellite 2 and 3) and dispersed repeats (LINE), but show no change at heavily methylated alpha-satellites. (b) Digestion by McrBC for primary breast tumors and breast cancer cell lines shows genomic demethylation at tandem (Satellite 2 and 3) and dispersed repeats (LINE). The same blot was repeatedly stripped and reprobed for the indicated regions. Digestions of DNA isolated from normal brain tissue are used as a reference control.

The available genome assemblies largely lack long tandem repeats, whose methylation status has been reported to be altered in mammary carcinoma [8]–[10]. The methylation status of these sequences was addressed by DNA blot hybridization after cleavage by methylation-sensitive restriction endonucleases or by the methylation-dependent McrBC complex. Of 41 primary breast cancers analyzed, 18 showed evidence of demethylation at Satellite 2, Satellite 3 and promoter regions of LINE-1 retrotransposon sequences as measured by increased resistance to McrBC cleavage, as did 7 of 10 breast cancer cell lines (Figure 2). Demethylation of alpha satellite DNA was not detected in primary breast tumors; this provided a control for completeness of digestion. The frequency of gross global demethylation observed in this sample was consistent with that of earlier studies [8]–[11]. Demethylation was not associated with clinical and pathological factors including tumor size, stage, grade, hormone receptor status, or lymph node positivity, which is also consistent with earlier studies [12].

**Figure 3:**
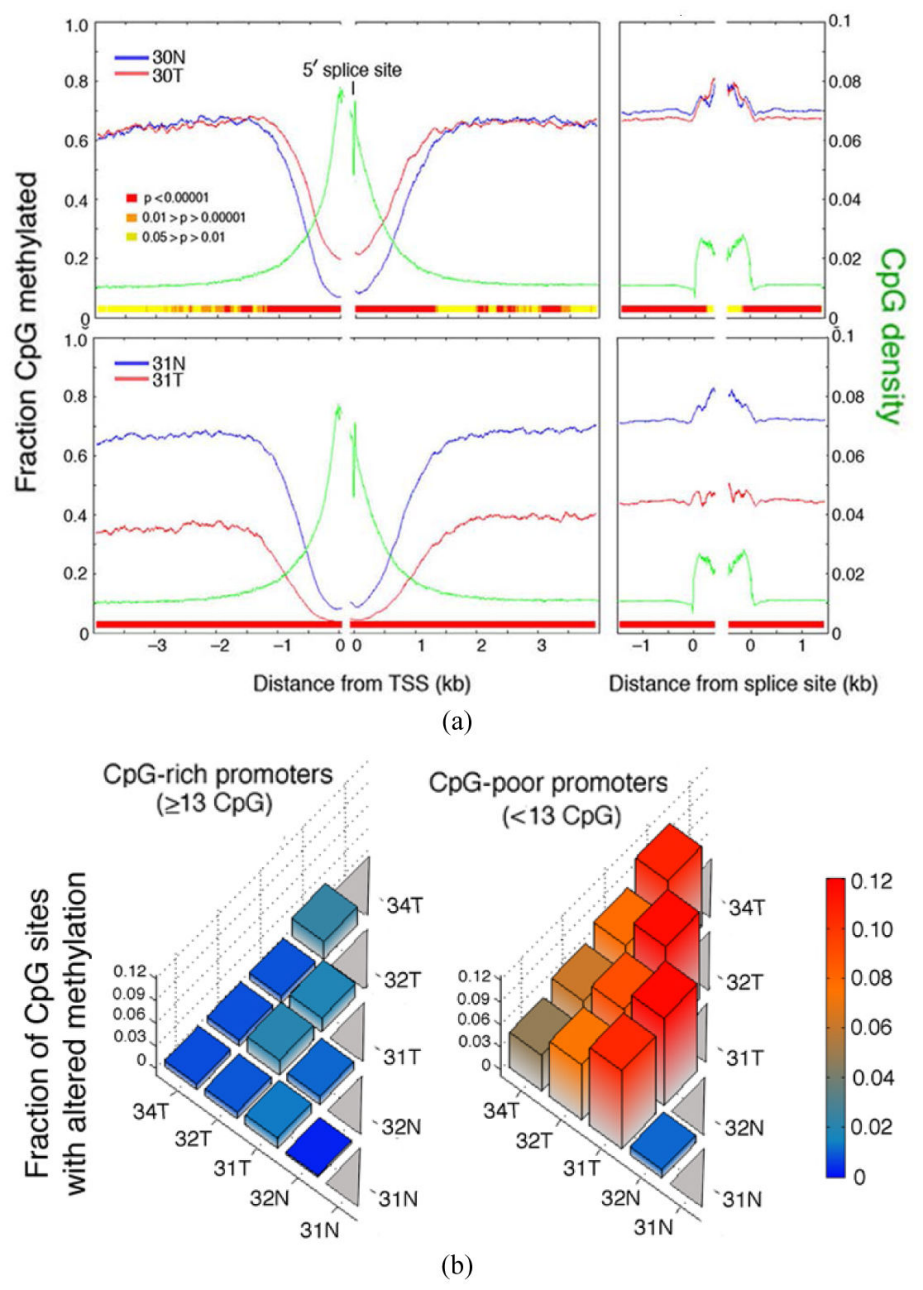
(a) CpG density (green) and the fraction of CpGs methylated are plotted as functions of distance to the TSS and 5′ splice site of the first exon (left panel) and from the 3′ and 5′ splice of internal exons (right panel). P-values for the differential methylation between the two samples were computed using a two-sample Kolmogorov-Smirnov test. (b) The fraction of significantly differentially methylated CpGs between each sample pair is plotted for all, CpG-rich, and CpG-poor promoters in the region −500 to +1 bp of the TSS. The distribution of the number of CpGs in this region was bimodal; a dividing line at 13 CpGs optimally separated the CpG-poor and -rich promoter classes [4]. Note that there was very little *de novo* methylation in the CpG-rich promoters that the greatest extent of *de novo* methylation in the CpG-poor promoters did not exceed ~1.6 methylated sites per promoter.

A metagene analysis of all RefSeq genes was conducted on the Methyl-MAPS data; the result is shown in Figure 3(a). As expected from the data in Figure 1, tumor 31T was found to be demethylated at promoters, introns, and exons relative to tumor 30T, but tumor 30T showed increased methylation density specifically in a region from −300 bp to +300 bp centered on the transcriptional start site (TSS); this was also true of tumor 32T (data not shown). In order to determine whether the increased methylation density in tumor 30T and 32T was due to large increases of methylation at a small number of promoters (as predicted by the epimutation model) or was due to small increases at large numbers of promoters, we analyzed the distribution of methylation changes found in promoters (Figure 3(b)). Two findings emerged from this analysis: First, few promoters showed increased methylation to >10% above control, and second, virtually all of the increased DNA methylation was in low-CpG promoters (<13 CpG sites) (Figure 3(b), right panel). The methylation increase did not exceed 1.6 methylated sites per promoter. No densely methylated CpG island promoter at any gene was detected in the cancer genomes.

**Figure 4:**
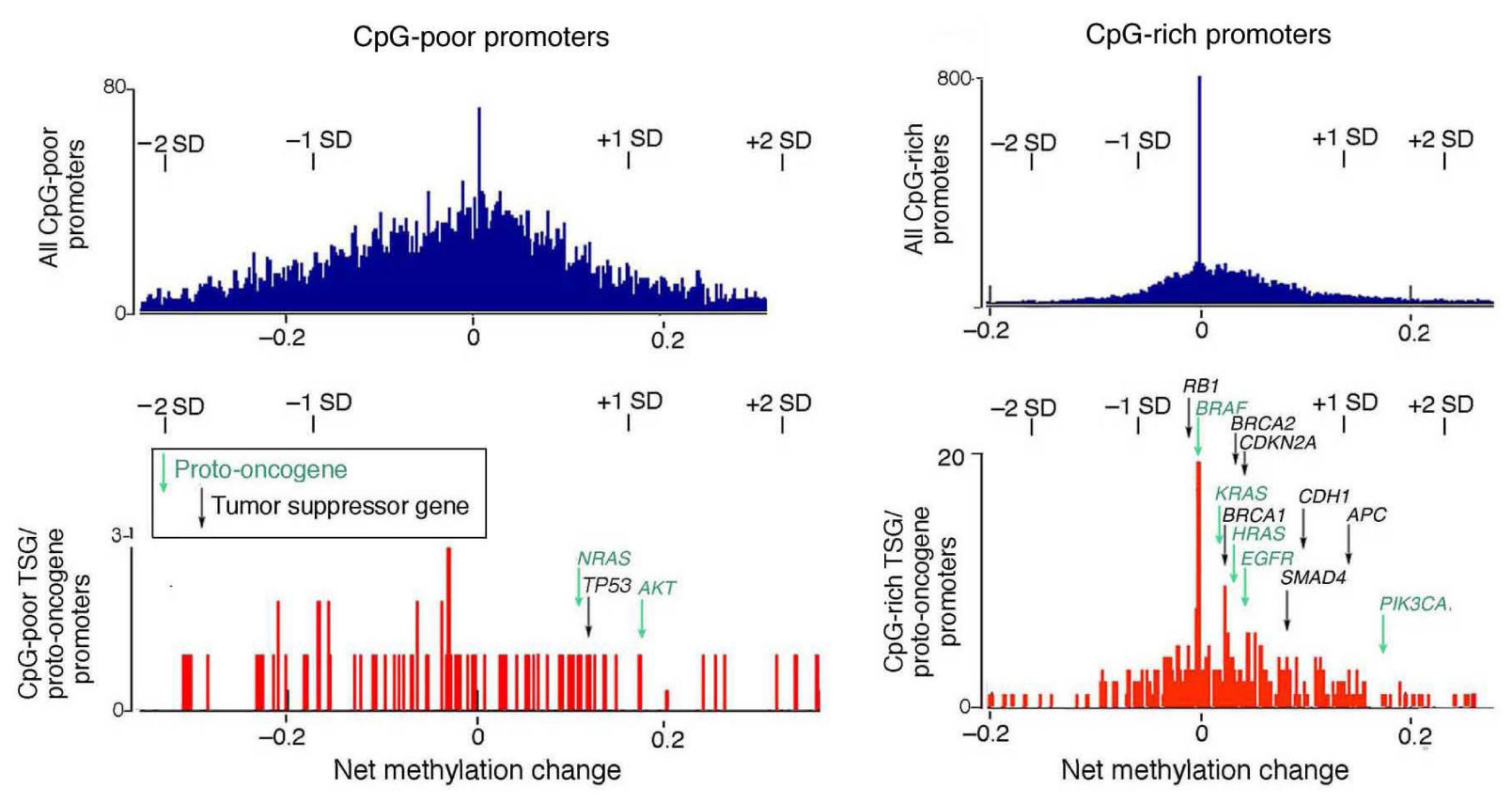
Histograms showing average methylation change between 32N and 32T for CpG-poor (left) and CpG-rich proximal promoters (right). Tumor suppressor genes (TSGs) are shown in black, proto- oncogenes in blue-green. The promoter region for each gene is defined as −500 to +1 bp of the TSS. The distribution of the number of CpGs in this region was bimodal; a dividing line at 13 CpGs optimally separated the CpG-poor and CpG-rich promoter classes [5]. Only CpGs with coverage of at least 10 in both samples were included. Upper panel shows methylation change across all promoters, noting one and two standard deviations (SD) from the mean. Lower panel shows distribution for genes known to be mutated in breast cancer (BrCa). A two-sample Kolmogorov-Smirnov test between the CpG-rich BrCa and all promoters yielded a p-value of 0.933, which is consistent with what would be expected if the two samples came from the same distribution. The mammary carcinoma gene list was compiled from Sanger COSMIC’s Biomart (COSMIC53 database [7]), selecting for all genes scored for mutations in two or more tumors and with primary site “breast”. Arrowed genes are the 20 genes most commonly mutated in mammary carcinoma [7].

The tumor suppressor genes (TSGs) that are most often mutated in mammary carcinoma (www.sanger.ac.uk/resources/databases/cosmic.html) were inspected for evidence of *de novo* promoter methyltion. The promoters were divided into CpG-poor and CpG-rich categories by the criteria described in [4] and analyzed separately. As shown in Figure 4, no tumor suppressor promoter examined showed evidence of increased methylation at a level of statistical significance of p < 0.05. The most heavily methylated promoter was that of proto-oncogene *PIK*3*CA*. The excess methylation in this gene was found to result from 2 heavily methylated reads among a total of 11 reads, 9 of which were unmethylated Figure S1. It is important to note that the extent of *de novo* methylation of proto-oncogenes (blue-green) was equivalent to that of tumor suppressors (black) (p = 0.933).

As shown in Supplementary Table S2, tumors 32T and 34T were triple negative and did not express *ER* (*ESR*1), *PR* (*PGR*) or *HER*2*/NEU* (*ERBB*2). *De novo* methylation has been implicated in silencing of each of these genes [13] [14], but as shown in Figure S2, there was no increased methylation of any of the four promoters of the *ESR*1 gene (three of which are very CpG-poor) or of the *PGR* or *ERBB*2 promoters. Thus the lack of expression of the *ESR*, *PGR*, and *ERBB*2 genes in the triple-negative tumors 32T and 34T cannot be attributed to increased methylation of their promoters.

**Figure 5:**
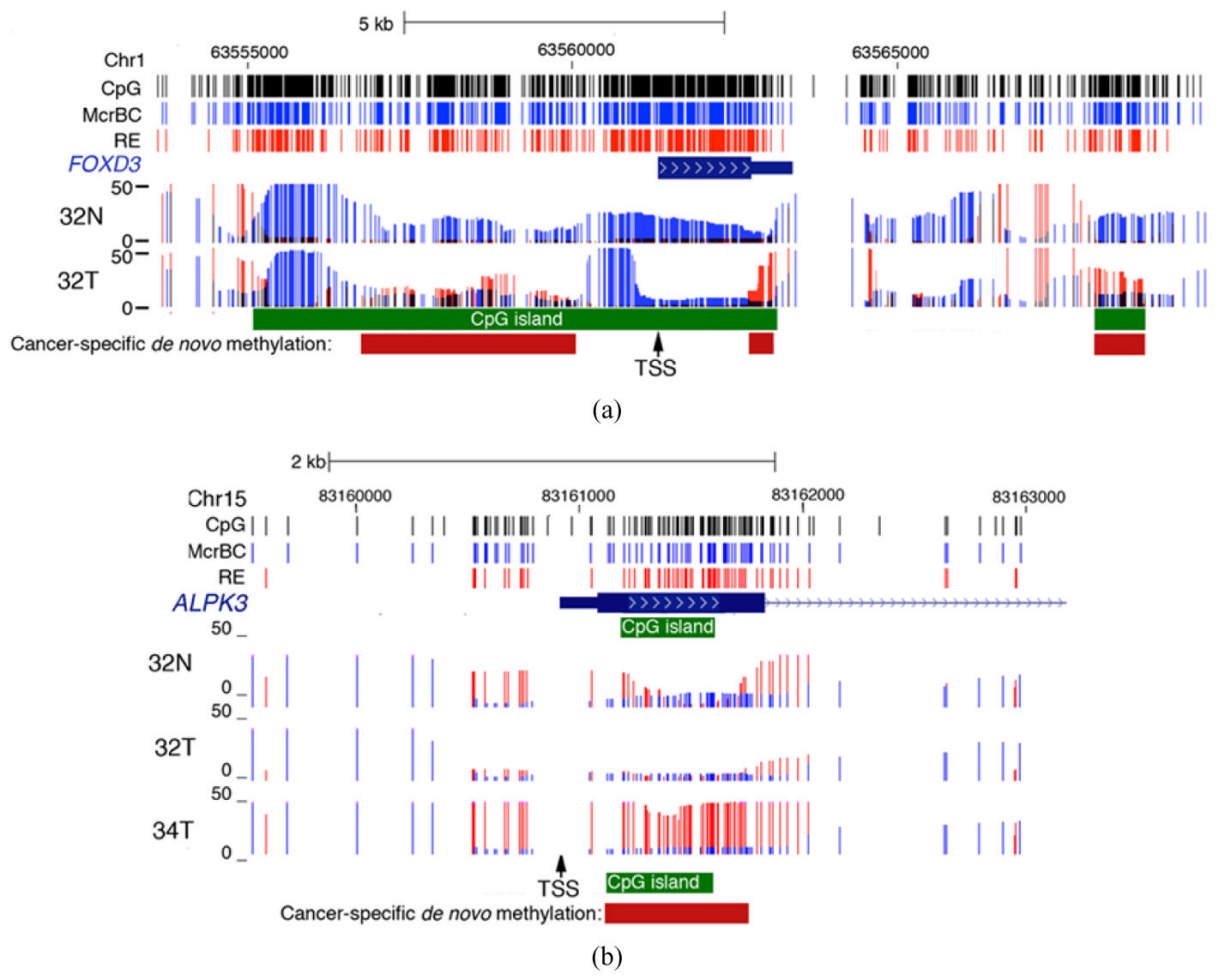

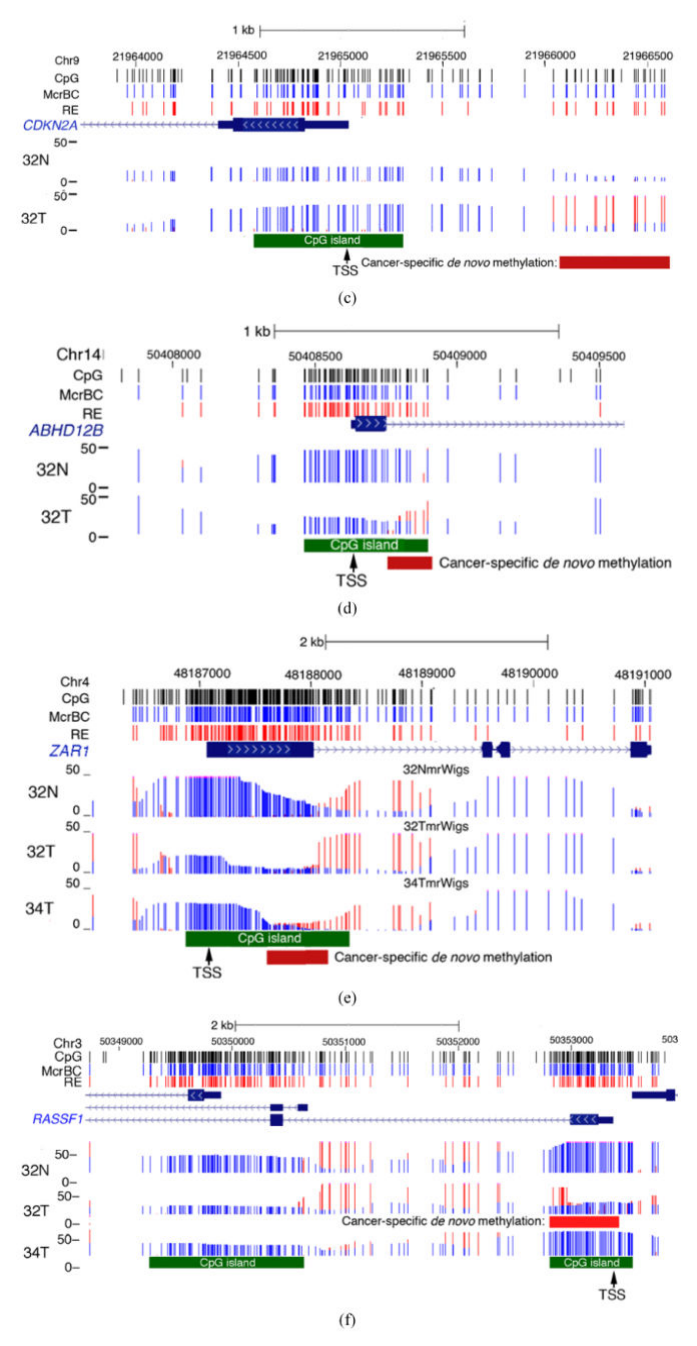

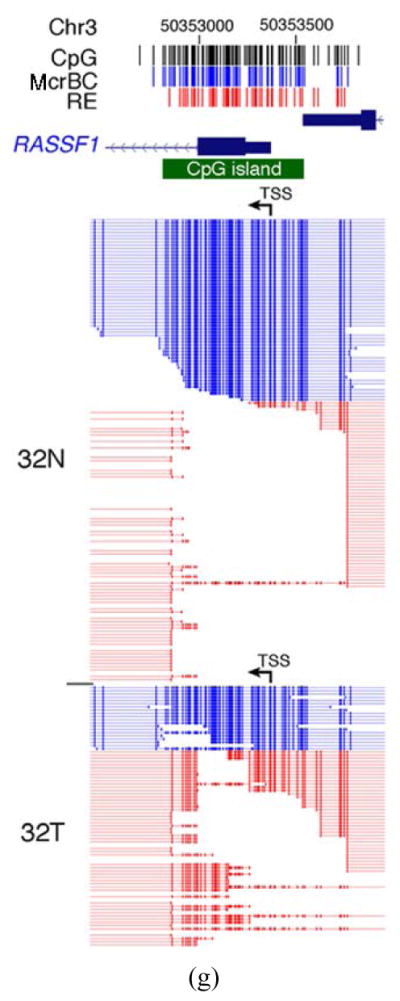
Examples of of cancer-specific methylation changes found in and around genes with sparing of proximal promoters. Methylation changes are sometimes upstream of the proximal promoter in CpG islands that are several kb upstream of the TSS (a) Or regions of low CpG density (b) Methylation changes found in CpG islands that overlap the TSS tend to occur away from the proximal promoter where methylation has been shown to affect gene silencing ((a), (c), (d), (e)). Coverage of McrBC (blue bars) and RE (red bars) at cleavable sites is indicated as an overlaid histogram. Tick marks both in tracks along the top of the figure indicate locations of individual RE and McrBC recognition sequences. (f) Cancer-specific methylation specifically in the CpG island of promoter 1 of RASSF1, with lack of methylation of promoters 2 and 3, which are the main sites of transcriptional initiation (Figure S2). (g) The individual sequences from the McrBC (blue) and RE (red) libraries for RASSF1 promoter 1. There are a greater number of unmethylated sequences at the TSS of promoter 1 than methylated sequences. It is unlikely that the observed methylation differences could silence RASSF1 expression, given that the major promoters are unmethylated.

Methyl-MAPS data were inspected for methylation changes in the vicinity of promoters that are unmethylated in control breast DNA. In many cases tumors showed focal *de novo* methylation of parts of CpG island sequences, but in nearly all cases the proximal promoter was found to be unaffected; five examples are shown in Figure 5. Cancer-specific *de novo* methylation of CpG island shores with sparing of proximal promoters has been previously reported [15]. In many cases *de novo* methylation anywhere in the vicinity of the promoter is referred to as promoter or CpG island methylation even when there is no reported methylation at the proximal promoter. It has long been known that DNA methylation represses transcription through its effects on the promoter and that methylation of other sequences has little or no effect [16] [17]. This is consistent with the finding that most sequences other than proximal promoters are usually methylated to greater or lesser extents in normal mammalian DNA [4].

The *RASSF*1 gene showed evidence of *de novo* methylation around a CpG island promoter in one of the three tumors examined, tumor 32T (Figure 5(f)) while a second alternative promoter was located in non-island sequence and a third was within a CpG island. The second and third promoters did not show evidence of differential methylation, and most transcripts from this locus originate at promoters two and three according to the representation of EST sequences (data not shown). Closer inspection of the methylated first promoter by examination of the individual sequence reads (Figure 5(g)) showed that while there was in fact methylation of sequences around the TSS of this promoter, the majority of sequences across this region were unmethylated. While this promoter would have been scored as methylated by most standard methods of methylation profiling, the presence of multiple unmethylated sequences at the TSS of the first promoter and the lack of differential methylation at the second and third promoters make it highly unlikely that the methylation at the first promoter could silence transcription of the *RASSF*1 gene.

## 3. Discussion

While it has long been held that tumor suppressor genes are inactivated by *de novo* methylation of their promoters during carcinogenesis, the hypothesis has remained unchallenged and unconfirmed. No methylation bio-marker of diagnostic effectiveness has yet resulted from this line of research, and the epimutation hypothesis has not formed the basis of new clinical applications. The results of whole genome methylation profiling in primary mammary carcinoma shown here indicate that the frequency of aberrant promoter methylation in this cancer is much lower than previously reported. These findings are consistent with a recent report in which gene silencing in mammary carcinoma is associated with an overall loss of methylation from affected genes and a gain of his-tone modifications usually associated with the transcriptionally inactive state [18].

Several factors could have led to an overestimation of the extent of promoter methylation in cancer. CpG islands have long been known to undergo *de novo* methylation during prolonged passage of cells in culture [18] [19], and tumor suppressor CpG islands have also been shown to be much more subject to *de novo* methylation in lines of cultured cells than in primary tumors [3]. Methodological factors also played a role. Bisulfite genomic sequencing is heavily biased in favor of methylated sequences [6], and methylated DNA immunoprecipitation (Me-DIP) is also biased in favor of methylated DNA because the anti-m^5^C antibody is biased against certain sequence compartments and cannot efficiently distinguish DNA fragments methylated at few versus many cytosines [20]. Another factor is the assumption that inspection of one or a few CpG sites flanking a given promoter will accurately reflect the methylation status of that promoter. As shown here, methylation patterns are highly heterogeneous at most CpG dinucleotides except for those within CpG-rich proximal promoters, which are almost uniformly unmethylated. It has long been known that the repressive effects of CpG methylation are exerted only when proximal promoter sequences are methylated [16], and in fact most CpG dinucleotides outside of promoters are methylated to variable extents in normal tissue DNA [4]. Our high coverage, whole-genome methylation profiles allowed the detailed examination of all known tumor suppressor genes; this analysis showed that tumors do not have an intrinsic propensity to methylate promoters. Furthermore, many genes have multiple transcriptional start sites, and a tumor suppressor gene can only be inferred to be silenced by *de novo* methylation if all of the start sites are both methylated in the entire cell population and all promoters are CpG-rich. None of the genes in our whole-genome analysis met either criterion.

We did confirm that genome-wide demethylation is a common event in mammary carcinoma [1]. Demethylation is quantitatively the most prominent methylation abnormality found in cancer genomes, but the mechanism and possible biological function of demethylation is obscure. We speculate that cancer-specific genome demethylation is a programmed response to a lack of growth control that kills incipient cancer cells through multiple effector pathways. First, genome demethylation induces apoptosis directly in non-stem cells. Embryonic stem cells that bear loss-of-function mutations in the *Dnmt*1 gene grow normally but die by apoptosis when induced to differentiate [21]. Demethylation to <~30% of wild type levels triggers apoptosis, and deletion of conditional alleles of *DNMT*1 in human HCT116 colorectal carcinoma cells triggers a mitotic catastrophe and apoptosis [22]. These data indicate that demethylation alone is sufficient to trigger apoptosis in non-ES cells. Some breast cancers (tumor 56T in Figure 2) and cell lines (Hs578T in Figure 2) have methylation levels reduced to be close to the point where apoptosis is induced. Second, the data presented here indicate that demethylation is likely to elicit an anti-tumor immune response. The promoters of cancer-testis (CT) antigen genes are normally heavily methylated and are not expressed except in male germ cells and in many cancers; the germ cells are protected from the immune system by the blood-testis barrier, but the expression of CT genes in tumors provokes an immune response. We suggest that demethylation and activation of the antigens encoded by CT genes is likely to facilitate the killing of cells that express the products of the demethylated CT genes, which is consistent with the frequent inflammation and anti-tumor immune responses seen in breast cancer [23] [24]. The release of demethylated DNA from lysed tumor cells will activate the innate immune response via the TLR9 pathway [25], which leads to the release of cytokines and the recruitment of immune effector cells that will augment the adaptive immune response against CT antigens.

We propose that a combination of anti-tumor adaptive and innate immune responses, together with demethylation-induced apoptosis, causes many incipient tumor cells to be killed before the tumor reaches detectable size. The interferon response, which is important in tumor defense, is frequently defective in cancer [26], and escape from immunosurveillance is a common feature of advanced carcinomas. Those tumors that grow to detectable size are those that have mutations that render them, which are insensitive to demethylation-induced immuno-surveillance or apoptosis.

The data presented here indicate that the epimutation hypothesis, under which the promoters of tumor suppressor genes undergo repressive *de novo* methylation as a result of unknown causes, may be much less significant in carcinogenesis than has been claimed. Genome-wide demethylation has been confirmed to occur in cancer genomes; this may. Demethylation may be a programmed response to a loss of growth control that activates a cell killing pathway that is part of tumor defense. Demethylation may have a tumor suppressive function; it may promote carcinogenesis, or it may be without significant effect. Identification of the mechanisms that drive demethylation in cancer would help to decide the issue.

## 4. Materials and Methods

### 4.1. Breast Cancer Tissue Bank

De-identified breast cancer tissue DNA and normal mammary gland tissue DNA (both classified as discarded material) were obtained from the Department of Pathology Tumor Bank Service at the Herbert Irving Comprehensive Cancer Center under a protocol judged exempt by the IRB of Columbia University. The study pathologist (H. H.) reviewed all tumors, pathological and laboratory parameters and clinical data. The use of these human tissue specimens in this study has been ruled exempt under NIH category 4.

### 4.2. Methyl-MAPS Library Preparation

Unmethylated and methylated compartments were obtained by limit DNA digestions as described in Rollins *et al*. [18] and Edwards *et al*. [4]. Paired-end libraries were prepared from the methylated and unmethylated DNA compartments by following an adaptation of Applied Biosystems’ SOLiD System Mate-paired Library Preparation Protocol.

### 4.3. Data Filtering and CpG Analysis

A custom perl script was written to parse the output files from the SOLiD system and filter sequences that did not have at least one restriction site (McrBC or RE, respectively) at the fragment ends. Since methylated (RE) and unmethylated (McrBC) compartments are sampled independently it is important to find the correct ratio of RE: McrBC fragments that represent the “true” distribution that would be obtained from a random sampling of the genome. This ratio can be determined numerically since if you fix the total number of McrBC + RE fragments, then using the ratio which matches the underlying “true” distribution should yield the maximum physical coverage. This ratio was estimated by finding the ratio of McrBC and RE fragments that maximized coverage on a subset of chromosomes (chromosomes 16 – 21). All McrBC and RE fragments were then overlapped with an indexed list of CpGs in hg18. The number of unmethylated observances at each CpG, *n_u_*, was set equal to the number of RE fragments that terminated at that CpG + the number of McrBC fragments containing that CpG in its interior (greater than 100 bp from the end). The number of methylated observances, *n_m_*, at a particular CpG was calculated as the sum of all RE fragments to which a particular CpG was interior. The coverage of a CpG at position *i* is then given as *C*(*i*) = *n_m_*(*i*) + *n_u_*(*i*) and the methylation score is calculated as *m*(*i*) = n*_m_*(*i*)/*C*(*i*).

CpG island, RepeatMasker, RefSeq gene data and other genomic annotation information was downloaded from the UCSC Genome Browser website. The average methylation score of a genomic element *e* is calculated as

$$\hat{m}(e)=\frac{1}{N(e)}\sum m(i)$$

where *N*(*e*) is the total number of CpGs in that element and where *C*(*i*) ≥ 5 and each CpG at position *i* are both McrBC and RE sites. All annotation and methylation data, indexed by CpG site was then stored in a MySQL database that could be used directly for calculations.

## Supplementary Material
