# Supplementary material for: Methylation Abnormalities in Mammary Carcinoma: The Methylation Suicide Hypothesis
Source: J Cancer Ther. Author manuscript; Available in PMC 2015 May 7. (PMC4423420; doi:10.4236/jct.2014.514131)
Supplement: Supplementary file 1 [file NIHMS681805-supplement-supplement_1.pdf]

Supplementary Data

Table S1. Histopathology of the mammary carcinomas subjected to whole-genome methylation analysis.

Tumor	Diagnosis	Tumor size (cm)	Cancer cells (%)	Grade	AJCC Stage	ER (ERBB2) (% stained)	RR (% stained)	HER2/ NEU	Classification
30T	Invasive carcinoma, mixed ductal and lobular feature, primarily lobular	5.5	80	2	3A	POS (65)	POS (85)	NEG	Luminal
31T	Invasive carcinoma, Edema of skin (peau d'orange) and ulceration of skin involved by cancer	14	85	3	3B	NEG (0)	NEG (0)	NEG	Triple negative
32T	Invasive carcinoma, ductal	1.7	90	3	3C	POS (50)	POS (50)	NEG	Luminal
34T	Invasive carcinoma	4.0	98	3	2	NEG (0)	NEG (0)	NEG	Triple negative

Table S2. Sequencing statistics for Methyl-MAPS analysis.

Sample	Total Paired End Reads	Total CpG Sites Tested	Physical Coverage
30N	8,593,791	60,529,032	4.9×
30T	24,367,296	181,306,252	13.3×
31N	12,407,641	94,392,993	8.2×
31T	32,923,635	186,806,278	15.2×
32N	74,588,780	738,511,384	54.5×
32T	83,343,938	675,567,405	51.0×
34T	82,981,578	788,744,498	60.1×
Sums:	319.206.659 reads	2,715,857,842 CpG sites	

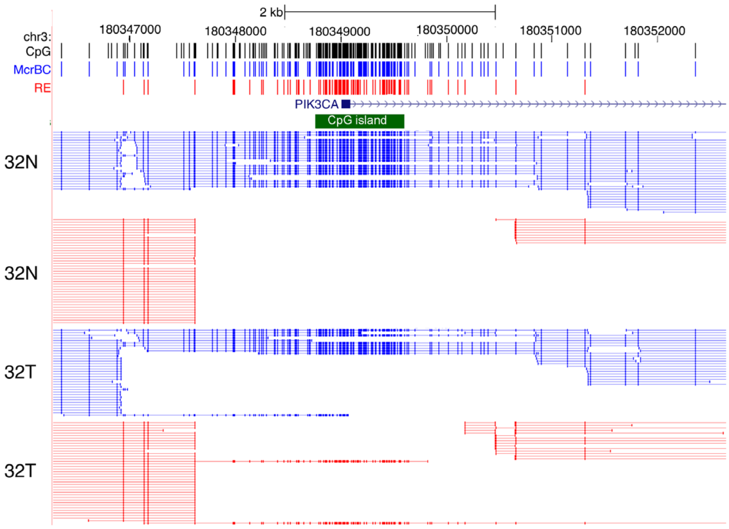

Figure S1. Methylation status of the 5' region of the tumor suppressor *PIK3CA*. In both normal (32N) and tumor (32T) DNA, the promoter region is unmethylated in almost the entire population; only two methylated alleles out of 11 in 32T are methylated.

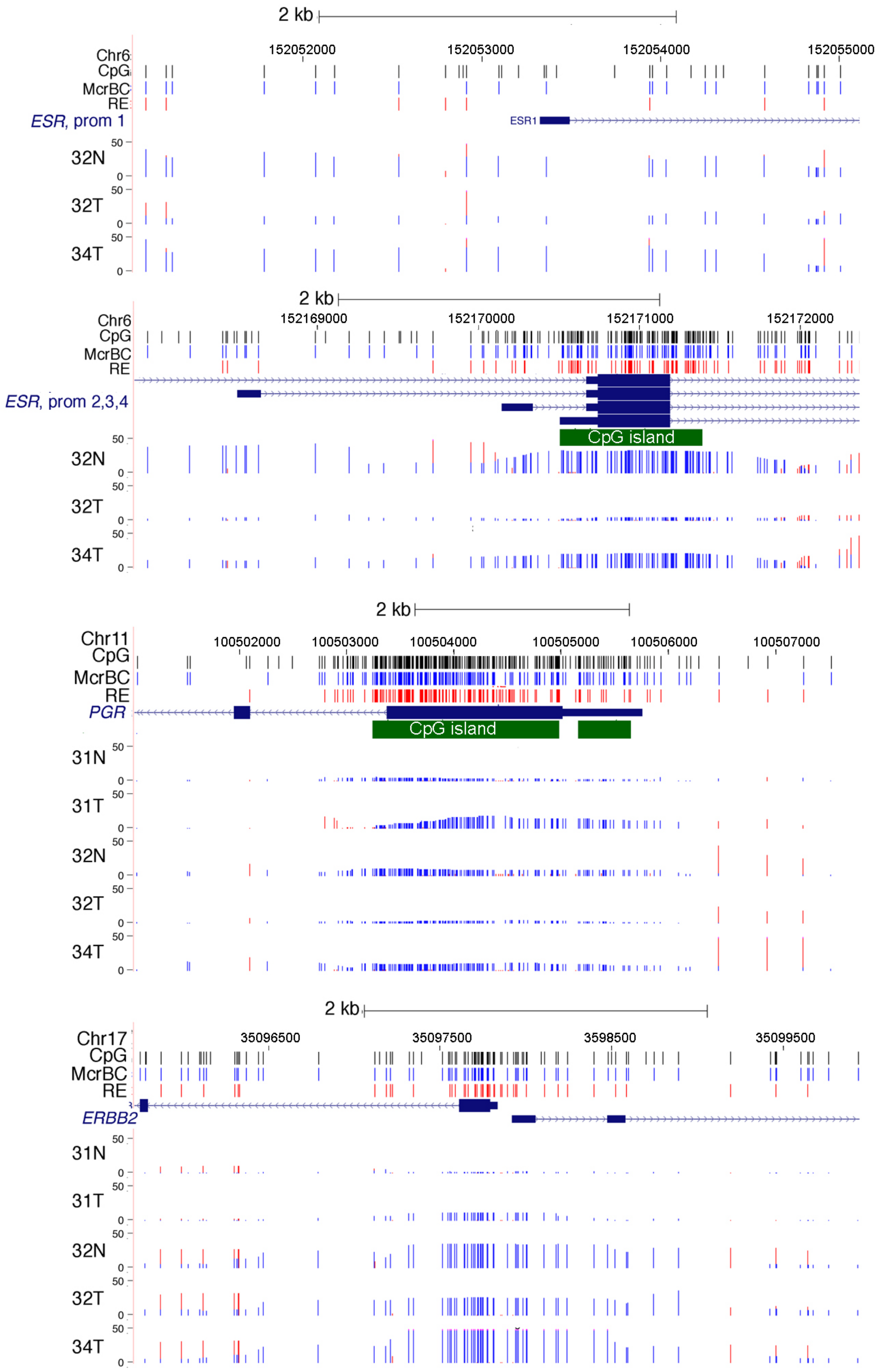

Figure S2. Methyl-MAPS data indicate that *ESR*1, *PGR*, and *ERBB*2 promoters are unmethylated in triple-negative mammary carcinomas 32T and 34T. All four alternative promoters for *ESR*1 (ESR prom 1, 2, 3, 4) are shown; only promoter 4 is within a CpG island. Coverage of McrBC (blue bars) and RE (red bars) at cleavable sites is indicated as an overlaid histogram. Tick marks in tracks along the top of the figure indicate locations of individual CpG dinucleotides and RE and McrBC recognition sequences.
